# Real-world multicenter assessment of sustained clinical outcomes after digital deep brain stimulation

**DOI:** 10.1038/s41746-025-02315-5

**Published:** 2026-01-14

**Authors:** Alireza Gharabaghi, Sergiu Groppa, Elena Casas, Alfons Schnitzler, Laura Muñoz-Delgado, Vicky L. Marshall, Jessica Karl, Lin Zhang, Ramiro Alvarez, Mary S. Feldman, Michael J. Soileau, Lan Luo, Benjamin L. Walter, Chengyuan Wu, Hong Lei, Damian M. Herz, Devyani Nanduri, Claudia A. Salazar, Corneliu Luca, Daniel Weiss

**Affiliations:** 1 Institute for Neuromodulation and Neurotechnology, Faculty of Medicine, University Hospital Tübingen (UKT), University Tübingen, Tübingen, Germany; 2 Center for Digital Health, Tübingen, Germany; 3 Center for Bionic Intelligence Tübingen Stuttgart (BITS), Tübingen, Germany; 4 German Center for Mental Health (DZPG), Tübingen, Germany; 5 Department of Neurology, University Hospital Homburg, Homburg, Germany; 6 Neurosurgery Department, La Princesa University Hospital, Madrid, Spain; 7 Department of Neurology, and Institute of Clinical Neuroscience and Medical Psychology, Medical Faculty and University Hospital Düsseldorf, Heinrich-Heine University Düsseldorf, Düsseldorf, Germany; 8 Unidad de Trastornos del Movimiento, Servicio de Neurología y Neurofisiología Clínica. Instituto de Biomedicina de Sevilla (IBiS), Hospital Universitario Virgen del Rocío/CSIC/Universidad de Sevilla, Sevilla, Spain; 9 Centro de Investigación Biomédica en Red sobre Enfermedades Neurodegenerativas (CIBERNED), Instituto de Salud Carlos III, Madrid, Spain; 10 Department of Neurology, Institute of Neurological Sciences, Queen Elizabeth University Hospital, Glasgow, UK; 11 Department of Neurological Sciences, Rush University, Chicago, IL USA; 12 Department of Neurology, University of California, Davis, Sacramento, CA USA; 13 Departamento de Neurociencias, Unidad de Enfermedades Neurodegenerativas, Servicio de Neurologia, Hospital Universitari Germans Trias I Pujol, Badalona, Spain; 14 Department of Neurology, Dartmouth-Hitchcock Medical Center, Lebanon, NH USA; 15 Texas Movement Disorder Specialists, PLLC, Georgetown, TX USA; 16 Department of Neurology, Beth Israel Deaconess Medical Center, Harvard Medical School, Boston, MA USA; 17 Department of Neurology, Indiana University School of Medicine, Indianapolis, IN USA; 18 Department of Neurological Surgery, Thomas Jefferson University, Philadelphia, PA USA; 19 Department of Neurology, University of Arizona, Tucson, AZ USA; 20 Abbott Neuromodulation, Austin, TX USA; 21 Department of Neurology, University of Miami, Miami, FL USA; 22 Center for Neurology, Department of Neurodegenerative Diseases, and Hertie Institute for Clinical Brain Research, University Tübingen, Tübingen, Germany; 23 Present Address: Feinberg School of Medicine, Northwestern University, Chicago, IL USA

**Keywords:** Diseases, Health care, Medical research, Neurology, Neuroscience

## Abstract

Remote, internet-based deep brain stimulation programming for Parkinson’s disease accelerates clinical benefits postoperatively by improving access to therapy adjustments compared to in-clinic optimization. After completion of the initial digital programming phase, we show that clinical outcomes, quality of life, and safety remain sustained over at least twelve months under routine care conditions. Embedding a randomized trial within a larger cohort study enables long-term, real-world evaluation, offering a scalable and pragmatic model for assessing complex digital interventions in routine clinical care. (NCT05269862 registered on 2022-03-08 and NCT04071847 registered on 2019-08-28).

## Introduction

Digital health interventions hold considerable promise for improving healthcare delivery by expanding access, enhancing personalization, and reducing resource demands^1^. Technologies such as mobile applications, remote monitoring platforms, and algorithm-guided tools are increasingly integrated into clinical care, yet their evaluation presents unique methodological challenges^2,3^. Unlike conventional interventions, digital therapeutics are often deployed in real-world settings where variability in user engagement, delivery contexts, and measurement fidelity can impact outcomes^4,5^. These complexities make it difficult for traditional randomized trial designs to fully capture the long-term effectiveness, sustainability, and generalizability of digital approaches^5,6^.

This challenge is particularly acute in device-assisted therapies, where access to specialist-guided care is essential^7^. Deep brain stimulation (DBS) is an established therapy for managing motor symptoms in Parkinson’s disease, but optimal outcomes depend on individualized programming and continued follow-up, typically conducted in specialized centers^8^. For many patients, these services are difficult to access due to travel burden, cost, or geographic limitations^9,10^. Digital models of care, such as remote DBS programming and virtual consultations, offer a promising solution^11–15^. However, little is known about their long-term clinical effectiveness and safety.

**Fig. 1:**
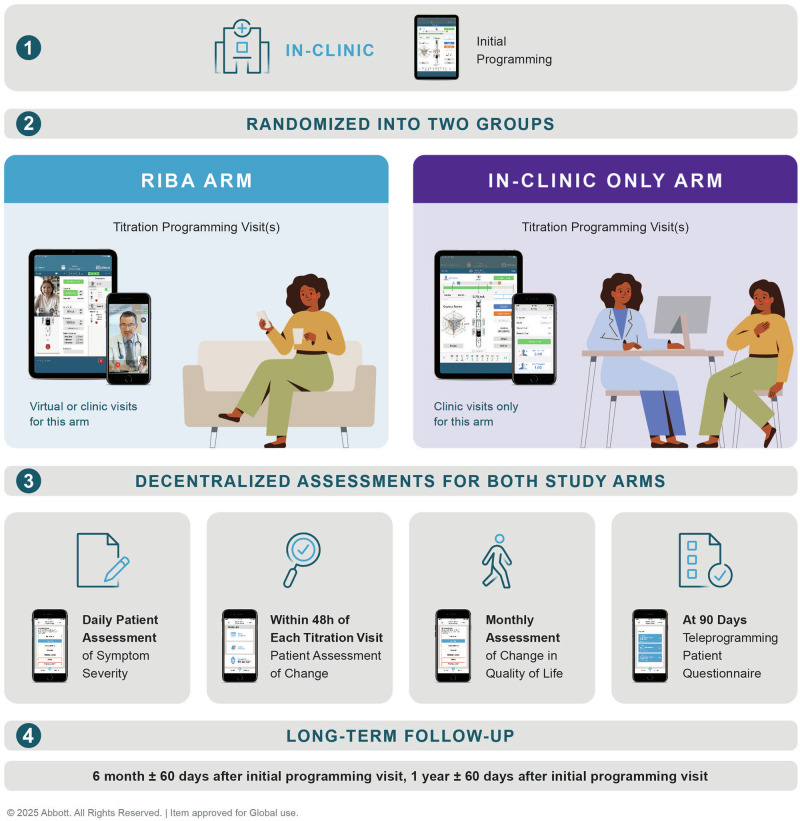
Overview of Data Collection^16^. Data collection was decentralized across both study arms. Initial programing was conducted in-clinic. Titration visits followed either in-clinic or remotely via virtual clinic (RIBA arm). Long-term follow-up occurred at 6 months ±60 days after initial programming, and 1-year ± 60 days after initial programming.

**Fig. 2:**
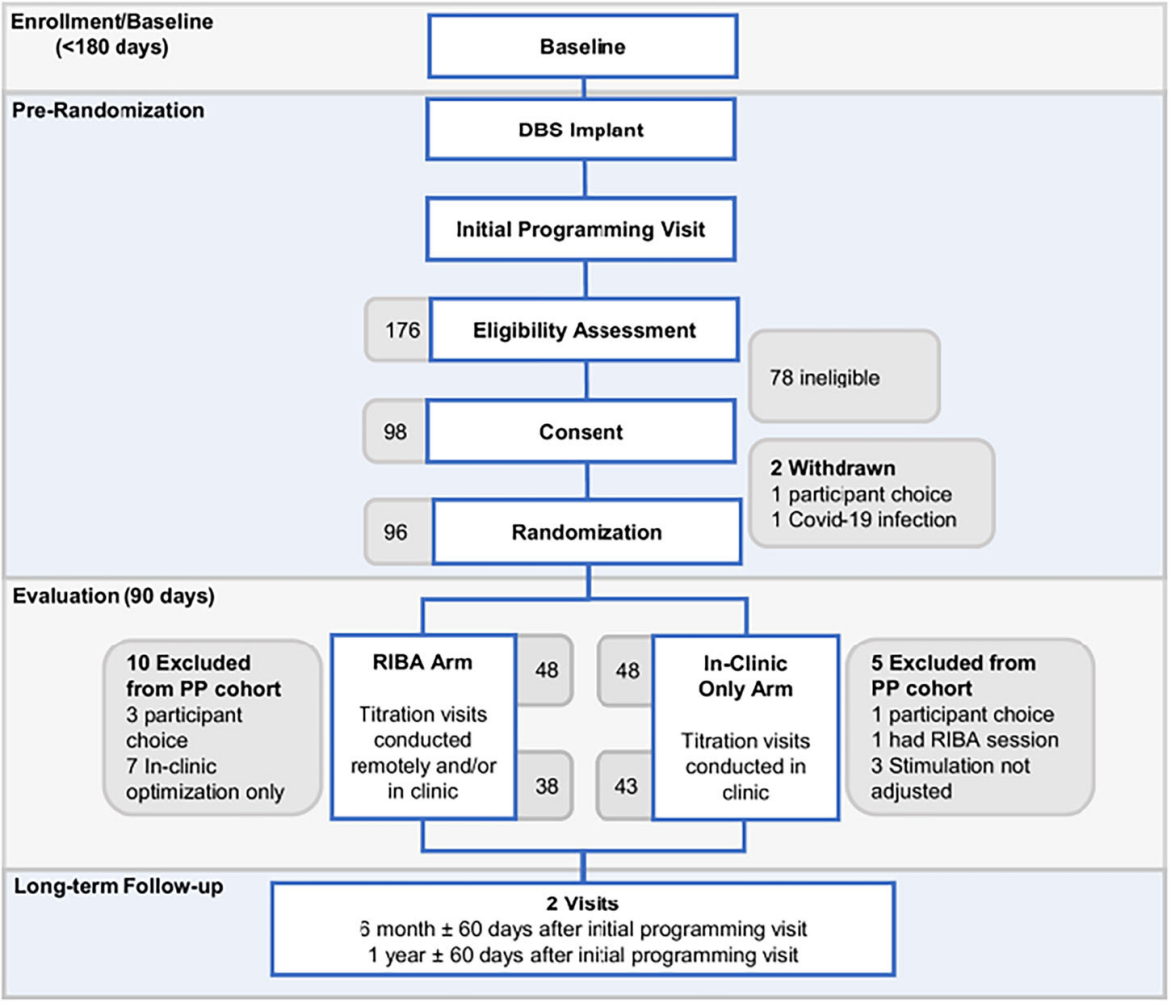
CONSORT Flow Diagram^16^. Study design overview^16^: Schematic representation of the hybrid ROAM-DBS/ADROIT study design. Following baseline enrollment, participants underwent DBS implantation, initial programming, and eligibility screening. After providing informed consent, patients were randomized into either the Virtual Clinic Arm (remote and/or in-clinic titration visits) or the In-Clinic Only Arm (titration visits conducted exclusively in clinic) for a 3-month randomized controlled trial (RCT) period. All participants were then followed in a long-term observational cohort (ADROIT) for five years with in-clinic follow-up visits. Here we report the findings at 6 and 12 months (±60 days) after the initial programming visit.

To address this gap, we implemented a multicenter, prospective hybrid study design by embedding a three-month randomized controlled trial (RCT) within a five-year observational cohort. The previously reported RCT compared remote, internet-based DBS programming via a virtual clinic platform with standard in-clinic follow-up, showing that virtual programming accelerated clinical improvement without compromising safety or patient satisfaction in the first three months after therapy initiation^16^. To evaluate the durability and generalizability of these effects, the embedded study design enabled ongoing follow-up of both groups under routine care conditions (Figs. 1 and 2). After the initial 3-month RCT phase, all participants had access to remote programming, with the choice between remote or in-clinic visits left to patients and/or their physicians, allowing for a real-world assessment of long-term outcomes (Table 1).

**Table 1:** Enrollment: Number of participants in each trial arm (in-clinic vs. virtual clinic) who completed follow-up visits at 3 months, 6 months, and 1 year after initial randomization

Visit Type	Number of Subjects Randomized	3 Months Follow-up Visit^a^	6 Months Follow-up Visit	1 Year Follow-up Visit
In-Clinic	48	43	39	38
Virtual Clinic	48	38	36	36

Retention remained high in both groups, with minimal attrition observed over the 12-month period, supporting the feasibility and acceptability of remote follow-up in routine clinical care. ^1^Either from electronic data capture (EDC) or home monitoring records.

^a^Either from electronic data capture (EDC) or home monitoring records.

This study was not designed to demonstrate replacement of in-clinic care by remote programming. After the RCT, remote programming became part of routine care, and withholding this approved option would not have been ethically justifiable. Patients with advanced Parkinson’s disease also require multidisciplinary management, including pharmacological adjustments and treatment of non-motor symptoms, and at most sites they attend routine in-clinic visits every six to twelve months independent of DBS therapy. Remote programming was therefore implemented as a complementary option rather than a substitute for in-clinic care.

## Results

Here, we report six- and twelve-month findings demonstrating sustained clinical benefits and similar improvements in outcomes, quality of life, and safety across both groups, supporting the ecological validity and scalability of this evaluation framework. Across all endpoints, outcomes remained consistently favorable and closely aligned supporting the clinical long-term equivalence of the remote and in-person care models used during the initial 3-month period. The observational phase was not intended to preserve strict group separation, since all patients had access to both modalities after three months. The purpose was to test whether the accelerated improvement observed during the titration phase with remote programming would persist once universal access was provided. The results confirm this, as outcomes converged and all patients followed a favorable trajectory at six and twelve months.

**Fig. 3:**
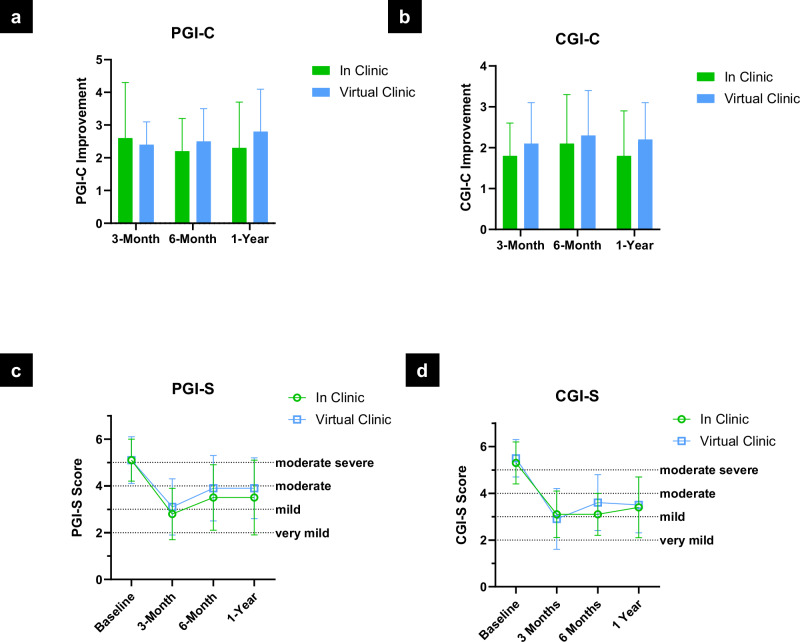
Patient and clinician scores. **a** Patient Global Impression (PGI) of Change: Mean PGI-C scores ± standard deviation are shown for patients at 3 months, 6 months, and 1 year after initial programming, comparing those who received in-clinic care to those who received remote, internet-based care via the virtual clinic platform. PGI-C is a 7-point patient-reported scale assessing overall improvement (1 = very much improved to 7 = very much worse). The number of patients (n) included at each time point is indicated in parentheses. Bracketed values represent 95% confidence intervals. Between-group differences were small and not statistically significant at any time point. ^1^By normal approximation. Additional information is included in Supplementary Table 1. **b** Clinician Global Impression (CGI) Change: Mean CGI-C scores at 3 months, 6 months, and 1 year as rated by clinicians for participants in the in-clinic and virtual clinic arms. The CGI-C is a 7-point scale evaluating overall clinical improvement (1 = very much improved to 7 = very much worse). Both care models demonstrated sustained clinician-rated improvement over 12 months. Differences between groups were small and not statistically significant at any timepoint. Data are shown as mean ± SD with corresponding 95% confidence intervals. Additional information is included in Supplementary Table 2. **c** Patient Global Impression (PGI) Severity: Mean PGI-S scores at baseline, 3 months, 6 months, and 1 year for participants in the in-clinic and virtual clinic arms, along with changes from baseline. Scores reflect patient-reported symptom severity on a 7-point scale (1 = not present to 7 = extremely severe). Both groups showed comparable improvements over time, with no statistically significant differences between groups across all timepoints. Data are presented as mean ± SD with corresponding 95% confidence intervals. Additional information is included in Supplementary Table 3. **d** Clinician Global Impression (CGI) Severity: Mean CGI-S scores assessed by clinicians at baseline, 3 months, 6 months, and 1 year for participants in the in-clinic and virtual clinic groups. The CGI-S is a 7-point scale evaluating overall symptom severity (1 = not present to 7 = extremely severe). Both groups showed sustained improvement from baseline, with similar reductions in perceived symptom severity over time. Differences between arms remained small and statistically non-significant across all timepoints. Data are presented as mean ± SD, with associated 95% confidence intervals. Additional information is included in Supplementary Table 4.

**Fig. 4:**
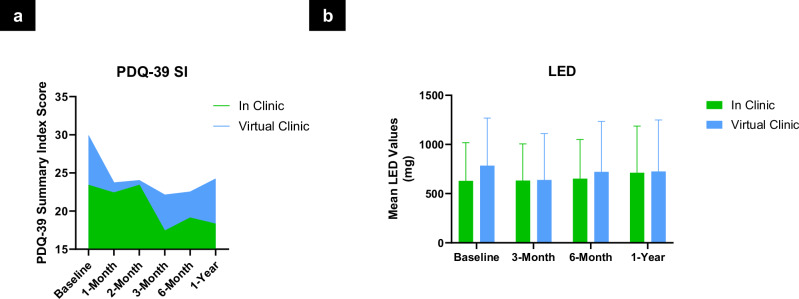
PDQ-39 and LED. **a** Parkinson’s Disease Questionnaire (PDQ-39) Summary Index (SI): Mean PDQ-39 Summary Index scores at baseline, and at 1, 2, 3, 6, and 12 months after treatment initiation in the in-clinic and virtual clinic groups. The PDQ-39 is a validated, patient-reported questionnaire assessing Parkinson’s disease-specific health status across eight domains, with higher scores indicating greater impairment and lower scores reflecting better quality of life. Both groups showed improvement from baseline over time. The virtual clinic group had slightly higher baseline impairment but demonstrated comparable or slightly greater reductions at follow-ups. Differences between groups were small and not statistically significant. Data are shown as mean ± SD, with 95% confidence intervals. Additional information is included in Supplementary Table 5. **b** Levodopa Equivalent Dose (LED): Mean LED values at baseline, 3, 6, and 12 months for the in-clinic and virtual clinic groups. LED provides a standardized method to quantify total dopaminergic medication load in patients with Parkinson’s disease. Both groups showed stable LED levels over the 12-month follow-up period, with modest increases observed at 12 months. Differences between groups were small and not statistically significant at most timepoints, although a higher mean LED was noted in the virtual clinic group throughout. Values are presented as mean ± SD, with 95% confidence intervals. ^1^The difference (mean comparison) is calculated by: (the average of virtual clinic – the average of in-clinic). Additional information is included in Supplementary Table 6.

Specifically, patient- and clinician-reported global improvement (PGI/CGI) remained favorable over time. Mean PGI change scores (Fig. 3a, Supplementary Table 1) were 2.3 ± 1.4 (in-clinic) and 2.8 ± 1.3 (virtual clinic), while CGI change scores (Fig. 3b, Supplementary Table 2) were 1.8 ± 1.1 and 2.2 ± 0.9, respectively, both consistent with sustained perceptions of improvement. Symptom severity improved comparably, with PGI severity scores (Fig. 3c, Supplementary Table 3) decreasing from 5.1 to 3.5 ± 1.6 in the in-clinic group and to 3.9 ± 1.3 in the virtual clinic group. CGI severity scores (Supplementary Table 4) mirrored these results, decreasing from 5.3 ± 0.9 to 3.4 ± 1.3 and from 5.5 ± 0.8 to 3.5 ± 1.2, respectively. The generalized mixed-effects model (Supplementary Tables 9–12) indicated significant longitudinal improvements in PGI-C and CGI-C (p-values for PGI-C and CGI-C were 0.04 and 0.0071, respectively), with no significant between-group differences (group effect p-values for PGI-C and CGI-C were 0.9839 and 0.5890, respectively). Quality of life, as measured by the PDQ-39 Summary Index (Fig. 4a, Supplementary Table 5), also improved in both groups (–4.3 ± 12.5 and –5.9 ± 13.1). The longitudinal analysis using a linear mixed-effects model (Supplementary Tables 13–14) revealed a significant improvement in PDQ-39 Summary Index scores from baseline (p = 0.0473), with no evidence of a group-by-time interaction (p = 0.9306). Motor function, assessed via MDS-UPDRS Part III (Supplementary Table 7), further improved in the MedON/StimON condition at twelve months, with similar scores between groups (15.3 ± 14.3 vs. 15.5 ± 9.4.5). Levodopa equivalent dose (LED) showed a trend toward reduction in the virtual clinic group (–64.8 ± 265.8 mg) and a slight increase in the in-clinic group (+99.1 ± 315.8 mg), though with high variability and overlapping confidence intervals (Fig. 4b, Supplementary Table 6).

Safety profiles were favorable and comparable across arms. No deaths were reported during the twelve-month period. Eight serious adverse device effects (SADEs) were reported in six patients. Three SADEs were related to the DBS procedure (all in the remote arm) and five to the impulse generator (four in the in-clinic arm and one in the remote arm). Two of the latter SADEs were related to DBS stimulation, both in the in-clinic group. One non-serious adverse device effect (ADE) in the in-clinic group was stimulation-related and resolved with re-programming. Healthcare utilization metrics (Supplementary Table 8), including emergency room visits (n = 13 per group) and hospitalization rates (n = 4 in-clinic, n = 3 virtual clinic), were similar across arms, indicating that remote care did not increase the burden on acute services. Protocol deviations (n = 57) were mostly related to scheduling or missing assessments and were evenly distributed without impact on outcomes or safety. None were related to the virtual clinic platform, further supporting its feasibility for routine use.

### Outcome measures

To assess the clinical impact and sustainability of remote DBS programming, a range of validated outcome measures were administered at multiple time points throughout the study. Assessments occurred at 3, 6, and 12 months after treatment initiation and covered patient-reported outcomes, clinician-rated evaluations, motor symptom severity, medication intake, quality of life, safety, and healthcare utilization. The 3-month timepoint corresponded to the end of the RCT phase, while the 6- and 12-month assessments were conducted during the subsequent open-label observational period.

Patient and clinician perspectives on treatment benefit and symptom burden were captured using the Patient Global Impression of Change (PGI-C) and Clinician Global Impression of Change (CGI-C), both 7-point scales rating perceived improvement from “very much improved” (1) to “very much worse” (7). Symptom severity was similarly rated using the Patient Global Impression of Severity (PGI-S) and Clinician Global Impression of Severity (CGI-S), ranging from “not present” (1) to “extremely severe” (7).

Levodopa Equivalent Dose (LED) was calculated to standardize and compare the total dopaminergic medication burden across participants, accounting for multiple medications with differing potencies.

Motor function was evaluated using Part III of the MDS-UPDRS at 6- and 12-months. This section consists of 33 item scores derived from 18 motor assessments. Each item is scored on a 5-point scale (0 = normal to 4 = severe), with higher cumulative scores indicating greater motor impairment.

To monitor health-related quality of life, the PDQ-39 was administered at 1, 2, 3, 6, and 12 months. This self-administered questionnaire evaluates eight dimensions of daily functioning (e.g., mobility, emotional well-being, cognition), with a Summary Index score reflecting the global impact of Parkinson’s disease on health status. Scores range from 0 to 100, with lower values indicating better quality of life.

Healthcare utilization and safety were monitored by documenting any emergency room visits or hospitalizations at 6 and 12 months. In addition, the incidence of device- or procedure-related serious adverse events (SAEs) was systematically recorded. SAEs were defined by standard regulatory criteria (e.g., hospitalization, death, permanent impairment). All reported safety events were adjudicated independently by a panel of three qualified clinicians.

## Discussion

These findings demonstrate that remote, internet-based DBS management can achieve sustained clinical outcomes over at least twelve months, maintaining improvements in global status, symptom burden, and quality of life without compromising safety or increasing acute care utilization. The remote programming platform allowed clinicians to perform the same range of adjustments as during in-clinic sessions, including amplitude, pulse width, frequency, and contact configuration. Sessions were conducted synchronously via secure video with real-time assessment of symptom response. In the randomized phase, no adjustments made remotely required later correction in clinic, supporting the clinical equivalence of both modalities for DBS optimization. Consistent with this, large-scale real-world implementation of remote DBS programming has also demonstrated safety, feasibility, and usability, with about 800 sessions successfully conducted shortly after clinical introduction^17^. Importantly, the early gains observed with virtual programming within the first three months were preserved at one year, suggesting that benefits are durable and not merely driven by initial novelty or user enthusiasm. This adds real-world evidence that digital interventions can perform on par with in-clinic models when carefully implemented and supported by infrastructure^18^.

Beyond clinical durability, this study addresses key barriers in digital health research by illustrating a practical and efficient hybrid trial design. By embedding the RCT within an existing observational study, we minimized resource demands, reduced trial burden, and captured long-term outcomes without parallel infrastructure. This approach is particularly well-suited for evaluating digital therapeutics, which often require adaptive, user-driven delivery in naturalistic settings^19^. It also enhances the persistent challenges of generalizability and scalability in digital medicine, while maintaining methodological rigor. As digital health solutions become increasingly personalized, embedded hybrid study designs offer a practical blueprint for evaluating long-term effectiveness and feasibility in real-world care^20,21^.

Nevertheless, several limitations merit consideration. The twelve-month phase was observational and not powered for detecting between-group differences, which may have limited the ability to uncover subtle effects. Programming modality (remote versus in-clinic) was not documented during the observational phase, since after the RCT remote programming became part of standard care. This limits long-term comparisons between strictly separated groups. However, the design reflects real-world clinical practice, where both modalities are available and cannot ethically be withheld. Patients with advanced Parkinson’s disease also undergo regular in-clinic visits for multidisciplinary management beyond DBS therapy, reinforcing that remote programming should be regarded as complementary rather than substitutive.

Participant characteristics, such as higher digital literacy or willingness to engage with virtual care, may also limit generalizability. The absence of formal blinding or sham control, while typical in digital trials, restricts interpretation of subjective endpoints. Finally, some outcome variables, including medication adjustments and unscheduled visits, may have been influenced by external clinical factors beyond the study protocol. Future studies should aim to replicate these findings in broader populations and more diverse care settings.

In conclusion, remote digital management of DBS therapy delivers sustained and clinically meaningful benefits that match traditional care models over the long term. The hybrid trial design employed here supports a scalable, pragmatic framework for evaluating complex digital interventions in chronic disease management. These results reinforce the value of embedding digital care models into structured real-world research, offering both a clinical and methodological foundation for future innovation in digital medicine.

## Methods

This study employed a prospective, multicenter hybrid design by embedding a three-month randomized controlled trial (RCT), which investigated remote internet-based adjustment of stimulation parameters (ROAM-DBS), within a larger post-market clinical follow-up cohort, which was designed to evaluate the long-term safety and effectiveness of DBS systems in real-world settings over a five-year period (ADROIT). Conducted across 17 clinical sites in the US and Europe, the trial adopted a decentralized structure, with most assessments and interventions conducted in participants’ home environments, consistent with ecological momentary assessment (EMA) principles. We obtained approval of the study protocol from Beth Israel Deaconess Medical Center Institutional Review Board, Dartmouth-Hitchcock Health Institutional Review Board, Rush University Medical Center Institutional Review Board, The Cleveland Clinic Institutional Review Board, Western Institutional Review Board-Copernicus Group (covering several US sites), Comité de Ética de La Investigación con Medicamentos del Hospital Universitario de la Princesa (covering all Spanish sites), Ethik-Kommission an der Medizinische Fakultät der Heinrich Heine Universität Düsseldorf, Ethik-Kommission bei der Landesärztekammer Rheinland-Pfalz, Ethik-Kommission an der Medizinische Fakultät der Eberhard-Karls-Universität und am Universitätsklinikum Tübingen, and the North of Scotland Research Ethics Committee. The authors complied with all relevant ethical regulations when conducting the study. Patients received detailed study information and signed an informed consent form before enrolling in the study. Both ROAM-DBS and ADROIT are registered on ClinicalTrials.gov (NCT05269862 registered on 2022-03-08 and NCT04071847 registered on 2019-08-28, respectively). The three-months results have been described previously^16^, here we report the twelve-months outcomes. Detailed methods have also been reported and are cited here where applicable^16^.

### Study enrollment and allocation

All ADROIT participants were screened for inclusion in the ROAM-DBS study. Eligible patients were adults (≥21 years) with Parkinson’s disease scheduled for de novo implantation of the Infinity DBS System with the NeuroSphere™ Virtual Clinic feature (Abbott, USA). Inclusion criteria required the ability, either personally or via caregiver support, to participate in remote care sessions, including adequate internet access and technical competence.

Patients were enrolled up to six weeks before implantation and at least one week before initial programming. Baseline assessments included demographics, disease duration, medication use, Movement Disorders Society Unified Parkinson’s Disease Rating Scale (MDS-UPDRS), and Parkinson’s Disease Questionnaire-39 (PDQ-39). All but three randomized patients were provided an iPhone™ and Apple Watch™ for decentralized collection of patient-reported outcomes (PROs) and exploratory remote monitoring of motor symptoms. Delays in device provision affected three patients at study onset.

Randomization for the three-months RCT phase occurred postoperatively in a 1:1 ratio using a computer-generated, block-randomized scheme (blocks of four), managed by an independent statistician and concealed from the analysis team. Patients were allocated sequentially, independent of patient characteristics or site discretion, minimizing allocation bias. After initial in-clinic programming, patients received ongoing DBS adjustments either in clinic (IC) or via remote internet-based adjustment (RIBA). Randomization after initial programming avoided bias in initial stimulation settings. Neither patients nor clinicians were blinded to treatment allocation. All procedures followed site-specific standards of care and clinical necessity. After three months, all patients received access to RIBA, and it was thereafter at the discretion of patients and/or their physicians to choose between remote programming or in-clinic visits, with both options available to participants from both arms.

### Statistical analysis

Statistical analysis was completed using SAS version 9.4 (SAS Institute Inc., Cary, NC, USA). Categorical data are presented as proportions and continuous variables are summarized with means and standard deviations (SD). 95% confidence intervals (CIs) are provided to compare intervention arms for non-powered endpoints. For longitudinal clinical outcomes (PGI-C, CGI-C, PDQ-39 Summary Index) we fitted linear mixed-effects models with fixed effects for randomized group (in clinic vs virtual clinic), time, the group-by-time interaction, and a random intercept for each participant. From these models, the improvement from baseline, between-group differences, and the interaction between group and time were examined. For the mixed-effects model analysis, PGI-C and GCI-C scores were dichotomized into a binary outcome (improved vs. not improved), with scores of or below 3 classified as improvement and scores of 4 or higher classified as no improvement. Figures were developed using GraphPad Prism (GraphPad Software, La Jolla, CA, USA).

### Inclusion & Ethics

The investigators involved in this study were all movement disorder specialists, with efforts made to balance representation and geographic heterogeneity. The study directly addressed two critical and commonly cited barriers to research participation: logistical constraints (such as time, caregiving responsibilities, and transportation) and the lack of clinical infrastructure in underserved areas. By utilizing the RIBA program and collecting PROs remotely, the study enabled participation without requiring clinic visits, thereby expanding access to populations traditionally underrepresented in research, including those in rural or economically disadvantaged communities. However, limited internet access remains a challenge, particularly among low-income and older adults, and continues to restrict broader inclusion. As cellular network coverage (e.g., Edge, 3 G, 4 G, and 5 G) continues to expand globally, the feasibility of remote DBS programming is expected to improve further. These findings underscore the potential of digital health solutions to reduce barriers to care and increase equity in clinical research and real-world treatment delivery.

## Supplementary information

Supplementary information

## Data Availability

The datasets generated and/or analyzed during the current study are not publicly available due to restrictions associated with Abbott’s policies but are available from the corresponding author on reasonable request. Due to the proprietary nature of the custom SAS macros used, the code cannot be shared publicly. Statistical analysis was completed using SAS version 9.4 (SAS Institute Inc., Cary, NC, USA). Figures were developed using GraphPad Prism (GraphPad Software, La Jolla, CA, USA).

## Code availability

Due to the proprietary nature of the custom SAS macros used, the code cannot be shared publicly. Statistical analysis was completed using SAS version 9.4 (SAS Institute Inc., Cary, NC, USA). Categorical data are presented as proportions and continuous variables are summarized with means and standard deviations (SD). 95% confidence intervals (CIs) are provided to compare intervention arms for non-powered endpoints. Figures were developed using GraphPad Prism (GraphPad Software, La Jolla, CA, USA).
