# Supplementary material for: Real-world multicenter assessment of sustained clinical outcomes after digital deep brain stimulation
Source: NPJ Digit Med. 2026 Jan 14;9:133. doi: 10.1038/s41746-025-02315-5 (PMC12881580; doi:10.1038/s41746-025-02315-5)
Supplement: Supplementary file 1 — Supplementary information [file 41746_2025_2315_MOESM1_ESM.pdf]

Supplementary Table 1. Patient Global Impression (PGI) of Change: Mean PGI-C scores ± standard deviation are shown for patients at 3 months, 6 months, and 1 year after initial programming, comparing those who received in-clinic care to those who received remote, internet-based care via the virtual clinic platform. PGI-C is a 7-point patient-reported scale assessing overall improvement (1 = very much improved to 7 = very much worse). The number of patients (n) included at each time point is indicated in parentheses. Bracketed values represent 95% confidence intervals. Between-group differences were small and not statistically significant at any time point. [1]By normal approximation.

	In-Clinic	Virtual Clinic	Difference [95% Confidence Interval][1]
Patients: 3 Months [95% Confidence Interval][1]	2.6 ± 1.7 (11) [1.5, 3.8]	2.4 ± 0.7 (9) [1.9, 3.0]	-0.2 [-1.4, 1.0]
Patients: 6 Months [95% Confidence Interval][1]	2.2 ± 1.0 (39) [1.8, 2.5]	2.5 ± 1.0 (36) [2.1, 2.8]	0.3 [-0.2, 0.8]
Patients: 1 Year [95% Confidence Interval][1]	2.3 ± 1.4 (38) [1.8, 2.7]	2.8 ± 1.3 (36) [2.4, 3.2]	0.5 [-0.1, 1.1]

Supplementary Table 2. Clinician Global Impression (CGI) Change: Mean CGI-C scores at 3 months, 6 months, and 1 year as rated by clinicians for participants in the in-clinic and virtual clinic arms. The CGI-C is a 7-point scale evaluating overall clinical improvement (1 = very much improved to 7 = very much worse). Both care models demonstrated sustained clinician-rated improvement over 12 months. Differences between groups were small and not statistically significant at any timepoint. Data are shown as mean ± SD with corresponding 95% confidence intervals.

	**In-Clinic**	**Virtual Clinic**	**Difference [95% Confidence Interval][1]**
Clinicians: 3 Months [95% Confidence Interval][1]	1.8 ± 0.8 (12) [1.3, 2.2]	2.1 ± 1.0 (10) [1.4, 2.8]	0.4 [-0.5, 1.2]
Clinicians: 6 Months [95% Confidence Interval][1]	2.1 ± 1.2 (39) [1.7, 2.5]	2.3 ± 1.1 (36) [1.9, 2.6]	0.2 [-0.3, 0.7]
Clinicians: 1 Year [95% Confidence Interval][1]	1.8 ± 1.1 (38) [1.5, 2.2]	2.2 ± 0.9 (35) [1.9, 2.5]	0.4 [-0.0, 0.9]

Supplementary Table 3. Patient Global Impression (PGI) Severity: Mean PGI-S scores at baseline, 3 months, 6 months, and 1 year for participants in the in-clinic and virtual clinic arms, along with changes from baseline. Scores reflect patient-reported symptom severity on a 7-point scale (1 = not present to 7 = extremely severe). Both groups showed comparable improvements over time, with no statistically significant differences between groups across all timepoints. Data are presented as mean ± SD with corresponding 95% confidence intervals.

	In-Clinic	Virtual Clinic	Difference [95% Confidence Interval][1]
Patients: Baseline [95% Confidence Interval][1]	5.1 ± 0.9 (43) [4.8, 5.3]	5.1 ± 1.0 (38) [4.8, 5.4]	0.0 [-0.4, 0.5]
Patients: 3 Months [95% Confidence Interval][1]	2.8 ± 1.1 (38) [2.5, 3.2]	3.1 ± 1.2 (34) [2.7, 3.6]	0.3 [-0.3, 0.8]
Patients: Difference b/w Baseline and 3 Months [95% Confidence Interval][1]	-2.2 ± 1.4 (38) [-2.7, -1.8]	-2.0 ± 1.5 (34) [-2.5, -1.5]	0.2 [-0.4, 0.9]
Patients: 6 Months [95% Confidence Interval][1]	3.5 ± 1.4 (39) [3.1, 4.0]	3.9 ± 1.4 (36) [3.4, 4.4]	0.4 [-0.3, 1.0]
Patients: Difference b/w Baseline and 6 Months [95% Confidence Interval][1]	-1.6 ± 1.6 (39) [-2.2, -1.1]	-1.3 ± 1.5 (36) [-1.8, -0.8]	0.4 [-0.3, 1.1]
Patients: 1 Year [95% Confidence Interval][1]	3.5 ± 1.6 (38) [2.9, 4.0]	3.9 ± 1.3 (36) [3.5, 4.3]	0.4 [-0.3, 1.1]
Patients: Difference b/w Baseline and 1 Year [95% Confidence Interval][1]	-1.6 ± 1.9 (38) [-2.2, -1.0]	-1.3 ± 1.4 (36) [-1.7, -0.8]	0.4 [-0.4, 1.2]

Supplementary Table 4. Clinician Global Impression (CGI) Severity: Mean CGI-S scores assessed by clinicians at baseline, 3 months, 6 months, and 1 year for participants in the in-clinic and virtual clinic groups. The CGI-S is a 7-point scale evaluating overall symptom severity (1 = not present to 7 = extremely severe). Both groups showed sustained improvement from baseline, with similar reductions in perceived symptom severity over time. Differences between arms remained small and statistically non-significant across all timepoints. Data are presented as mean ± SD, with associated 95% confidence intervals.

	**In-Clinic**	**Virtual Clinic**	**Difference [95% Confidence Interval][1]**
Clinicians: Baseline<br>[95% Confidence Interval][1]	5.3 ± 0.9 (43)<br>[5.0, 5.6]	5.5 ± 0.8 (35)<br>[5.2, 5.8]	0.2<br>[-0.2, 0.6]
Clinicians: 3 Months<br>[95% Confidence Interval][1]	3.1 ± 1.0 (12)<br>[2.5, 3.7]	2.9 ± 1.3 (10)<br>[2.0, 3.8]	-0.2<br>[-1.2, 0.9]
Clinicians: Difference b/w Baseline and 3 Months<br>[95% Confidence Interval][1]	-2.1 ± 0.8 (12)<br>[-2.6, -1.6]	-2.6 ± 1.3 (10)<br>[-3.6, -1.6]	-0.5<br>[-1.6, 0.5]
Clinicians: 6 Months<br>[95% Confidence Interval][1]	3.1 ± 0.9 (39)<br>[2.8, 3.3]	3.6 ± 1.2 (36)<br>[3.2, 3.9]	0.5<br>[0.0, 1.0]
Clinicians: Difference b/w Baseline and 6 Months<br>[95% Confidence Interval][1]	-2.3 ± 1.1 (39)<br>[-2.6, -1.9]	-2.0 ± 1.2 (33)<br>[-2.4, -1.6]	0.3<br>[-0.3, 0.8]
Clinicians: 1 Year<br>[95% Confidence Interval][1]	3.4 ± 1.3 (38)<br>[2.9, 3.8]	3.5 ± 1.2 (35)<br>[3.1, 3.9]	0.1<br>[-0.5, 0.7]
Clinicians: Difference b/w Baseline and 1 Year<br>[95% Confidence Interval][1]	-1.9 ± 1.6 (38)<br>[-2.5, -1.4]	-2.1 ± 1.3 (33)<br>[-2.6, -1.6]	-0.1<br>[-0.8, 0.5]

Supplementary Table 5. Parkinson's Disease Questionnaire (PDQ-39) Summary Index: Mean PDQ-39 Summary Index scores at baseline, and at 1, 2, 3, 6, and 12 months after treatment initiation in the in-clinic and virtual clinic groups. The PDQ-39 is a validated, patient-reported questionnaire assessing Parkinson's disease-specific health status across eight domains, with higher scores indicating greater impairment and lower scores reflecting better quality of life. Both groups showed improvement from baseline over time. The virtual clinic group had slightly higher baseline impairment but demonstrated comparable or slightly greater reductions at follow-ups. Differences between groups were small and not statistically significant. Data are shown as mean ± SD, with 95% confidence intervals.

	In-Clinic	Virtual Clinic	Difference [95% Confidence Interval][1]
Baseline<br>[95% Confidence Interval][1]	23.4 ± 12.8 (39)<br>[19.3, 27.5]	29.9 ± 13.6 (38)<br>[25.4, 34.3]	-6.5<br>[-12.4, -0.5]
1 Months<br>[95% Confidence Interval][1]	22.4 ± 14.1 (34)<br>[17.4, 27.3]	23.7 ± 14.7 (31)<br>[18.3, 29.1]	-1.4<br>[-8.5, 5.8]
Difference b/w Baseline and 1 Months<br>[95% Confidence Interval][1]	-3.9 ± 14.3 (31)<br>[-9.1, 1.4]	-6.8 ± 13.1 (31)<br>[-11.6, -2.0]	2.9<br>[-4.0, 9.9]
2 Months<br>[95% Confidence Interval][1]	23.4 ± 13.9 (34)<br>[18.6, 28.3]	24.0 ± 15.3 (30)<br>[18.3, 29.8]	-0.6<br>[-8.0, 6.7]
Difference b/w Baseline and 2 Months<br>[95% Confidence Interval][1]	-1.5 ± 13.5 (32)<br>[-6.4, 3.3]	-4.7 ± 14.7 (30)<br>[-10.2, 0.7]	3.2<br>[-4.0, 10.4]
3 Months<br>[95% Confidence Interval][1]	17.4 ± 11.5 (32)<br>[13.3, 21.5]	22.1 ± 12.4 (29)<br>[17.4, 26.8]	-4.7<br>[-10.9, 1.4]
Difference b/w Baseline and 3 Months<br>[95% Confidence Interval][1]	-6.2 ± 12.0 (30)<br>[-10.7, -1.7]	-6.9 ± 14.4 (29)<br>[-12.4, -1.4]	0.7<br>[-6.2, 7.6]
6 Months<br>[95% Confidence Interval][1]	19.1 ± 12.0 (35)<br>[15.0, 23.2]	22.5 ± 16.7 (35)<br>[16.8, 28.2]	-3.4<br>[-10.3, 3.6]
Difference b/w Baseline and 6 Months<br>[95% Confidence Interval][1]	-3.9 ± 12.9 (32)<br>[-8.5, 0.8]	-7.8 ± 16.9 (35)<br>[-13.6, -2.0]	3.9<br>[-3.4, 11.2]
1 Year<br>[95% Confidence Interval][1]	18.3 ± 12.1 (34)<br>[14.1, 22.6]	24.2 ± 13.5 (34)<br>[19.5, 28.9]	-5.9<br>[-12.1, 0.3]
Difference b/w Baseline and 1 Year<br>[95% Confidence Interval][1]	-4.3 ± 12.5 (33)<br>[-8.7, 0.1]	-5.9 ± 13.1 (34)<br>[-10.5, -1.3]	1.6<br>[-4.6, 7.9]

Supplementary Table 6. Levodopa Equivalent Dose (LED): Mean LED values at baseline, 3, 6, and 12 months for the in-clinic and virtual clinic groups. LED provides a standardized method to quantify total dopaminergic medication load in patients with Parkinson's disease. Both groups showed stable LED levels over the 12-month follow-up period, with modest increases observed at 12 months. Differences between groups were small and not statistically significant at most timepoints, although a higher mean LED was noted in the virtual clinic group throughout. Values are presented as mean ± SD, with 95% confidence intervals. [1]The difference (mean comparison) is calculated by: (the average of virtual clinic – the average of in-clinic).

	In-Clinic	Virtual Clinic	Difference (Mean Comparison)[1]
Baseline<br>[95% Confidence Interval][1]	629.8 ± 388.6 (31)<br>[487.2, 772.3]	784.3 ± 484.1 (29)<br>[600.2, 968.4]	-154.5<br>[-382.7, 73.6]
3 Months<br>[95% Confidence Interval][1]	633.4 ± 372.2 (30)<br>[494.4, 772.4]	693.9 ± 470.3 (28)<br>[511.6, 876.3]	-60.5<br>[-285.1, 164.0]
Difference b/w Baseline and 3 Months<br>[95% Confidence Interval][1]	-9.2 ± 228.6 (30)<br>[-94.6, 76.1]	-96.9 ± 287.6 (28)<br>[-208.5, 14.6]	87.7<br>[-49.8, 225.3]
6 Months<br>[95% Confidence Interval][1]	652.3 ± 398.7 (31)<br>[506.1, 798.5]	721.6 ± 512.9 (28)<br>[522.7, 920.4]	-69.2<br>[-311.2, 172.7]
Difference b/w Baseline and 6 Months<br>[95% Confidence Interval][1]	27.7 ± 275.4 (30)<br>[-75.2, 130.5]	-69.3 ± 265.8 (28)<br>[-172.4, 33.7]	97.0<br>[-45.4, 239.4]
1 Year<br>[95% Confidence Interval][1]	712.1 ± 474.2 (29)<br>[531.8, 892.5]	726.1 ± 522.9 (28)<br>[523.3, 928.9]	-14.0<br>[-279.3, 251.4]
Difference b/w Baseline and 1 Year<br>[95% Confidence Interval][1]	99.1 ± 315.8 (28)<br>[-23.4, 221.5]	-64.8 ± 265.8 (28)<br>[-167.9, 38.3]	163.9<br>[7.4, 320.4]

Supplementary Table 7. Motor Scores: Mean MDS-UPDRS Part III motor scores at baseline, 6 months, and 1 year across different medication/stimulation states (ON/ON, OFF/OFF, OFF/ON, ON/OFF) for both in-clinic and virtual clinic groups. Data reflect motor function improvements following DBS therapy under various treatment conditions. Not all combinations were collected at every timepoint. Values are presented as mean ± SD with number of participants (N) indicated per condition. NA = Not Assessed. Lower scores indicate better motor performance

	In-Clinic (N=41)			Virtual Clinic (N=38)		
**Medication/ Stimulation**	**Baseline**	**6 Months**	**1 Year**	**Baseline**	**6 Months**	**1 Year**
ON/ON	NA	17.5 ± 11.3 (37)	15.3 ± 13.4 (33)	NA	19.4 ± 13.2 (35)	15.5 ± 9.4 (35)
OFF/OFF	43.3 ± 16.9 (36)	NA	51.9 ± 19.1 (14)	46.8 ± 15.8 (36)	NA	53.3 ± 17.5 (10)
OFF/ON	NA	30.0 ± NA (1)	25.1 ± 17.1 (17)	NA	NA	29.1 ± 12.0 (11)
ON/OFF	21.8 ± 11.0 (41)	NA	NA	23.3 ± 10.2 (36)	NA	NA

Supplementary Table 8: Health resource utilization: Summary of emergency room (ER) visits and hospitalizations reported during the 12-month follow-up period for patients initially randomized to in-clinic versus virtual clinic programming. Events are presented as absolute counts and percentages of affected patients (n/N) within each group. Overall healthcare utilization was similar between groups, with 17 events in the in-clinic group and 16 in the virtual clinic group, indicating no increased burden on acute care services from remote management.

Visit	In-Clinic Events	In-Clinic (%(n/N))	Virtual Clinic Events	Virtual Clinic (%(n/N))
ER Visits	13	14.0% (6/43)	13	13.2% (5/38)
Hospitalization	4	7.0% (3/43)	3	5.3% (2/38)
Total	17	9	16	5

Supplementary Table 9. Fixed Effects Estimates for PGI-C Scores from Generalized Mixed-Effects Model: Estimates and p-values from the generalized mixed-effects model for PGI-C scores. The model included fixed effects for randomized group (Virtual vs. In-Clinic), timepoints (3-month, 6-month, 1-year), and their interactions, with Virtual group and 3-month as reference categories. A random intercept for each participant was included to account for repeated measures.

**Effect**	**Estimate**	**P-value**
Intercept	2.1972	0.0400
Randomized Group (In-Clinic)	-1.0986	0.3805
Timepoint (6-Month)	0.1054	0.9289
Timepoint (1-Year)	-0.9734	0.3842
Interaction Term (In-Clinic x 6-Month)	1.8165	0.2393
Interaction Term (In-Clinic x 1-Year)	1.5124	0.2695

Note: The reference groups are Virtual for randomized groups and 3-month for timepoint.

Supplementary Table 10. PGI-C Type III Tests for Fixed Effects: Type III tests for fixed effects from the linear mixed-effects model for PGI-C scores. P-values from Type III tests evaluating the overall significance of randomized group, timepoint, and group-by-time interaction in the linear mixed-effects model.

Effect	P-value
Randomized Group	0.9839
Timepoint	0.0673
Interaction Term	0.4705

Supplementary Table 11. Fixed Effects Estimates for CGI-C Scores from Generalized Mixed-Effects Model: Estimates and p-values from the generalized mixed-effects model for CGI-C scores. The model included fixed effects for randomized group (Virtual vs. In-Clinic) and timepoints (3-month, 6-month, 1-year), with Virtual group and 3-month as reference categories.

**Effect**	**Estimate**	**P-value**
Intercept	2.9686	0.0071
Randomized Group (In-Clinic)	0.3410	0.5890
Timepoint (6-Month)	-0.4892	0.6630
Timepoint (1-Year)	-0.3330	0.7699

Note: The reference groups are Virtual for randomized groups and 3-month for timepoint. The generalized mixed models with interaction terms did not converge; hence, a model without interaction terms is shown here.

Supplementary Table 12. CGI-C Type III Tests for Fixed Effects: P-values from Type III tests evaluating the overall significance of randomized group and timepoint in the linear mixed-effects model. The fixed effects include randomized group (Virtual vs. In-Clinic) and timepoint (3-month, 6-month, 1-year).

**Effect**	**P-value**
Randomized Group	0.5890
Timepoint	0.8999

Supplementary Table 13. Fixed Effects Estimates for PDQ-39 Scores from the Linear Mixed-Effects Model: Estimates and p-values for fixed effects, including randomized group (Virtual vs. In-Clinic), timepoints (1-month [reference], 2-month, 3-month, 6-month, 1-year), and group-by-time interaction terms. The reference categories are Virtual for randomized group and 1-month for timepoint.

**Effect**	**Estimate**	**P-value**
Intercept	-4.4028	0.0473
Randomized Group (In-Clinic)	1.9922	0.5238
Timepoint (2-Month)	0.1300	0.9457
Timepoint (3-Month)	-2.2362	0.2397
Timepoint (6-Month)	-1.4186	0.4387
Timepoint (1-Year)	-1.1159	0.5441
Interaction Term (In-Clinic x 2-Month)	0.7714	0.7738
Interaction Term (In-Clinic x 3-Month)	-1.0767	0.6936
Interaction Term (In-Clinic x 6-Month)	0.1229	0.9629
Interaction Term (In-Clinic x 1-Year)	-1.3113	0.6204

Note: The reference groups are Virtual for randomized groups and 1-month for timepoint.

Supplementary Table 14. PDQ-39 Type III Tests for Fixed Effects: P-values from Type III tests evaluating the overall significance of randomized group, timepoint, and group-by-time interaction in the linear mixed-effects model.

Effect	P-value
Randomized Group	0.5169
Timepoint	0.1100
Interaction Term	0.9306

Section/topic	No	CONSORT 2025 checklist item description	Reported on page no.
**Title and abstract**			
Title and structured abstract	1a	Identification as a randomised trial	4
	1b	Structured summary of the trial design, methods, results, and conclusions	4-10
**Open science**			
Trial registration	2	Name of trial registry, identifying number (with URL) and date of registration	3, 9
Protocol and statistical analysis plan	3	Where the trial protocol and statistical analysis plan can be accessed	10-11
Data sharing	4	Where and how the individual de-identified participant data (including data dictionary), statistical code and any other materials can be accessed	10-11
Funding and conflicts of interest	5a	Sources of funding and other support (eg, supply of drugs), and role of funders in the design, conduct, analysis and reporting of the trial	9-11
	5b	Financial and other conflicts of interest of the manuscript authors	10-11
**Introduction**			
Background and rationale	6	Scientific background and rationale	4-5
Objectives	7	Specific objectives related to benefits and harms	N/A
**Methods**			
Patient and public involvement	8	Details of patient or public involvement in the design, conduct and reporting of the trial	N/A
Trial design	9	Description of trial design including type of trial (eg, parallel group, crossover), allocation ratio, and framework (eg, superiority, equivalence, non-inferiority, exploratory)	4-5, 8-10
Changes to trial protocol	10	Important changes to the trial after it commenced including any outcomes or analyses that were not prespecified, with reason	N/A
Trial setting	11	Settings (eg, community, hospital) and locations (eg, countries, sites) where the trial was conducted	9
Eligibility criteria	12a	Eligibility criteria for participants	9-10
	12b	If applicable, eligibility criteria for sites and for individuals delivering the interventions (eg, surgeons, physiotherapists)	N/A
Intervention and comparator	13	Intervention and comparator with sufficient details to allow replication. If relevant, where additional materials describing the intervention and comparator (eg, intervention manual) can be accessed	N/A
Outcomes	14	Prespecified primary and secondary outcomes, including the specific measurement variable (eg, systolic blood pressure), analysis metric (eg, change from baseline, final value, time to event), method of aggregation (eg, median, proportion), and time point for each outcome	5-10, Supplemental Tables 1-7
Harms	15	How harms were defined and assessed (eg, systematically, non-systematically)	5-10, Supplemental Table 8
Sample size	16a	How sample size was determined, including all assumptions supporting the sample size calculation	4-5, 9-10
	16b	Explanation of any interim analyses and stopping guidelines	N/A
Randomisation:			
Sequence generation	17a	Who generated the random allocation sequence and the method used	9-10

	17b	Type of randomisation and details of any restriction (eg, stratification, blocking and block size)	9-10
			**Reported on page no.**
Allocation concealment mechanism	18	Mechanism used to implement the random allocation sequence (eg, central computer/telephone; sequentially numbered, opaque, sealed containers), describing any steps to conceal the sequence until interventions were assigned	9-10
Implementation	19	Whether the personnel who enrolled and those who assigned participants to the interventions had access to the random allocation sequence	9-10
Blinding	20a	Who was blinded after assignment to interventions (eg, participants, care providers, outcome assessors, data analysts)	10-11
	20b	If blinded, how blinding was achieved and description of the similarity of interventions	8-11
Statistical methods	21a	Statistical methods used to compare groups for primary and secondary outcomes, including harms	9-10
	21b	Definition of who is included in each analysis (eg, all randomised participants), and in which group	9-11
	21c	How missing data were handled in the analysis	6, 9-10
	21d	Methods for any additional analyses (eg, subgroup and sensitivity analyses), distinguishing prespecified from post hoc	N/A
**Results**			
Participant flow, including flow diagram	22a	For each group, the numbers of participants who were randomly assigned, received intended intervention, and were analysed for the primary outcome	9-11
	22b	For each group, losses and exclusions after randomisation, together with reasons	N/A
Recruitment	23a	Dates defining the periods of recruitment and follow-up for outcomes of benefits and harms	6-7, 9-11
	23b	If relevant, why the trial ended or was stopped	N/A
Intervention and comparator delivery	24a	Intervention and comparator as they were actually administered (eg, where appropriate, who delivered the intervention/comparator, how participants adhered, whether they were delivered as intended (fidelity))	9-11
	24b	Concomitant care received during the trial for each group	N/A
Baseline data	25	A table showing baseline demographic and clinical characteristics for each group	15-16
Numbers analysed, outcomes and estimation	26	For each primary and secondary outcome, by group:<br>● the number of participants included in the analysis<br>● the number of participants with available data at the outcome time point<br>● result for each group, and the estimated effect size and its precision (such as 95% confidence interval)<br>● for binary outcomes, presentation of both absolute and relative effect size	15-16
Harms	27	All harms or unintended events in each group	Supplemental Table 8
Ancillary analyses	28	Any other analyses performed, including subgroup and sensitivity analyses, distinguishing pre-specified from post hoc	N/A
**Discussion**			
Interpretation	29	Interpretation consistent with results, balancing benefits and harms, and considering other relevant evidence	7-8
Limitations	30	Trial limitations, addressing sources of potential bias, imprecision, generalisability, and, if relevant, multiplicity of analyses	7-8

Citation: Hopewell S, Chan AW, Collins GS, Hróbjartsson A, Moher D, Schulz KF, et al. CONSORT 2025 Statement: updated guideline for reporting randomised trials. BMJ. 2025; 388:e081123.
https://dx.doi.org/10.1136/bmj-2024-081123

© 2025 Hopewell et al. This is an Open Access article distributed under the terms of the Creative Commons Attribution License (https://creativecommons.org/licenses/by/4.0/), which permits unrestricted use, distribution, and reproduction in any medium, provided the original work is properly cited.

*We strongly recommend reading this statement in conjunction with the CONSORT 2025 Explanation and Elaboration and/or the CONSORT 2025 Expanded Checklist for important clarifications on all the items. We also recommend reading relevant CONSORT extensions. See www.consort-spirit.org.
